# Fabrication of Functional Polyurethane/Rare Earth Nanocomposite Membranes by Electrospinning and Its VOCs Absorption Capacity from Air

**DOI:** 10.3390/nano7030060

**Published:** 2017-03-11

**Authors:** Jun Cong Ge, Nag Jung Choi

**Affiliations:** Division of Mechanical Design Engineering, Chonbuk National University, 567 Baekje-daero, Jeonjusi 561-756, Jeollabuk-do, Korea; freedefeng@naver.com

**Keywords:** electrospinning, polyurethane, rare earth, nanocomposites, VOCs

## Abstract

Volatile organic compounds (VOCs) are a source of air pollution and are harmful to both human health and the environment. In this study, we fabricated polyurethane/rare earth (PU/RE) composite nanofibrous membranes via electrospinning with the aim of removing VOCs from air. The morphological structure of PU/RE nanofibrous mats was investigated using field emission scanning electron microscopy (FE-SEM), fourier transform infrared spectroscopy (FTIR), and X-ray diffraction (XRD) experimental analyses. A certain amount of RE (up to 50 wt. % compared to PU pellets) nanoparticles (NPs) could be loaded on/into PU fibers. The tensile strength of PU/RE nanofibrous membranes decreased slightly with the increasing RE powder content. The PU nanofiber containing 50 wt. % RE powder had the smallest fiber diameter of 356 nm; it also showed the highest VOC absorption capacity compared with other composite membranes, having an absorption capacity about three times greater than pure PU nanofibers. In addition, all of the PU/RE nanofibrous membranes readily absorbed styrene the most, followed by xylene, toluene, benzene and chloroform. Therefore, the PU/RE nanofibrous membrane can play an important role in removing VOCs from the air, and its development prospects are impressive because they are emerging materials.

## 1. Introduction

Volatile organic compounds (VOCs) are numerous, varied, and ubiquitous, and they are one of the sources of air pollution. VOCs can be categorized according to their chemical structures: alkanes, aromatic hydrocarbons, esters, aldehydes, alcohols, ethers, and amides [1]. There are about 300 different kinds of VOCs; most day-to-day activities lead to the production of VOCs, such as driving, cooking, building, decorating, painting, and even face-painting [2,3,4]. A high concentration of VOCs can easily lead to acute poisoning, lightheadedness, headache, dizziness, coughing, nausea, vomiting, and liver poisoning, and can even induce a coma. Benzene and formaldehyde are the most common carcinogenic and highly toxic pollutants in VOCs and they can quickly damage the human respiratory system [1]. Therefore, it is important to effectively reduce the content of VOCs in the air, and finding a way to control and reduce the content of VOCs in air has attracted attention for a long time.

One area of interest includes the development of both VOC detection and control sensor technology [5,6,7,8,9], using the large surface area and large pore volume, among other advantages, of activated carbon to adsorb the VOCs [10,11,12,13,14,15]. Activated carbon is the most common adsorption method currently used. However, using activated carbon as an adsorption medium is less economic than that of composite nanofibrous materials. Furthermore, the adsorption capacity of the activated carbon decreases with high pressure drops over the adsorbent bed or after initial carbon fouling. In general, activated carbon regeneration is very difficult, except when using specialized methods (steam desorption) [16]. On the other hand, there has also been research regarding the development of new composite materials (such as nanocomposite thin films produced via electrospinning) to absorb VOCs [17,18,19,20]. Socholten et al. [16] and Kim et al. [19,21] reported that adsorption and desorption of VOCs by electrospun nanofibrous membranes was faster than that of conventional activated carbon.

Electrospinning is a fiber production method that provides a straightforward yet versatile approach for the convenient preparation of continuous fibers [22,23,24,25]. It has been widely applied to various fields, such as artificial skin, bandages, bulletproof clothing, battery electrolytes, sensors, and hierarchically structured fibrous composites [26,27,28,29]. The composite nanofibrous materials, which were fabricated using electrospinning technology, are more cost-effective, easy to use and versatile for the removal of VOCs [19,20,21].

Rare earth (RE) materials, ‘the Vitamins of Modern Industry’, play an important role in industrial development. Smelting and separation of RE raw materials can be accomplished and used for energetic materials, luminescent materials, grinding materials, environmental protection materials, and permanent magnetic materials [30]. In recent years, RE has been an emerging material for the removal of VOCs, and it contains a series of metal oxides, such as Ce_2_O_3_, CeO_2_, La_2_O_3_, Pr_2_O_3_, Nd_2_O_3_, Sm_2_O_3_, Er_2_O_3_ and so on. Some of these oxides are widely used in glassmaking, catalysts, ceramics and metallurgy [31]. More importantly, many researchers have reported that some of the individual rare earth elements can curb VOC pollution [32,33,34,35]. However, the RE elements combined with other materials, especially the nanocomposite fibrous membranes produced via electrospinning, still have not been studied on the removal characteristics of the VOCs.

Therefore, in this study, we investigated the combined characteristics of RE and polyurethane (PU) and their VOC adsorption capacity using the electrospinning technique. The morphology and components and mechanical properties were thoroughly investigated. A certain amount (up to 50 wt. % compared to PU pellets) of RE nanoparticles (NPs) could be loaded on/into PU fibers. The PU nanofiber containing 50 wt. % RE powder had the highest VOC absorption capacity, about three times greater than that of pure PU nanofibers. The as-fabricated PU/RE nanofibrous membranes are promising facile, practical materials for the removal of VOCs from air in our environment.

## 2. Experimental Details

### 2.1. Materials

Polyurethane (PU) pellets (Estane^®^ Skythane^®^ X595A-11) were purchased from Lubrizol Advanced Materials, Inc., Cleveland, OH, USA, and were used as the polymeric matrix. RE powder were purchased from ZIBO WEIJIE RARE EARTH CO., LTD, Zibo, China. *N*,*N*-Dimethylformamide (DMF) was purchased from Showa Chemical Co., Ltd., Tokyo, Japan. Extra pure methyl ethyl ketone (MEK, 2-butanone) was purchased from Junsei Chemical Co., Ltd, Tokyo, Japan. In VOCs absorption experiments, five different kinds of gases (styrene, xylene, chloroform, benzene, and toluene) (purity 99.9%, AR grade) were analyzed.

### 2.2. Fabrication of Composite Nanofibrous Membranes

**Figure 1:**
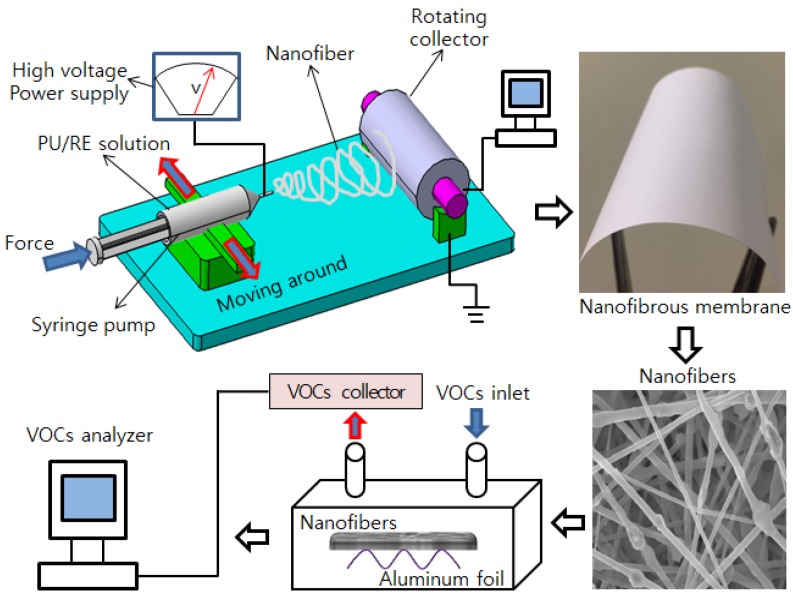
Schematic diagram of the electrospinning setup and volatile organic compounds (VOCs) absorption experiment.

The fabrication process of PU/RE nanofibrous membranes is given as follows. First, the PU pellets were dried (constant temperature: 80 °C) for about 3 h in a dry oven before dissolving them in the solvent. Second, 10 wt. % PU pellets were dissolved in a DMF/MEK (50:50 by weight) mixing solution, using a magnetic stirrer dissolved for 12 h at room temperature. Then 0, 10, 30, and 50 wt. % (compared to PU pellets) RE powders were added into above the solution as electrospun precursors via ultrasonication for 2 h. The process of electrospinning is shown in Figure 1. The electrospun temperature and relative humidity are constantly controlled at 23 ± 2 °C and 40%–50%, respectively. All PU/RE spinning solutions were electrospun at 15 kV of high-voltage electricity with an 18 cm tip-to-collector distance, the rotating speed of the rotating collector was 650 rpm, and the solution feed rate was 1 mL/h [19,20,21]. After electrospinning, the composite PU/RE nanofibrous membranes were dried (constant temperature: 80 °C) for 12 h in the vacuum oven. The PU nanofibers containing 0, 10 wt. %, 30 wt. % and 50 wt. % of RE powder are denoted as PU, PU/RE-10, PU/RE-30 and PU/RE-50, respectively.

### 2.3. Characterization

The morphology of the PU/RE nanofibrous membranes was observed using field emission scanning electron microscopy (FE-SEM, JIB-4601F, JEOL Ltd., Tokyo, Japan). The viscosity and conductivity of the spinning solutions were measured by an electric conductivity meter (EC meter cm-40G, Hyogo, Japan) and a viscometer (Brookfield LVDH-II) at room temperature. N_2_ adsorption-desorption isotherms were examined at 77 k by an ASAP 2020 physisorption analyzer (Micromeritics Co., Norcross, GA, USA). The mechanical properties of the PU/RE nanofibrous membranes were measured by a universal testing machine. Fiber diameters were measured with the ImageJ software on 50 random filaments. The Fourier transform infrared (FT-IR, Spectrum GX, PerkinElmer, Lnc., San Francisco, CA, USA) spectra of as-prepared PU/RE nanofibrous membranes were obtained using a Paragon 1000 spectrometer. An X-ray diffraction (XRD) analysis was carried out using a Multipurpose High Performance X-ray Diffractometer (X’ pert Powder, PANalytical, Eindhoven, The Netherlands).

### 2.4. VOCs Absorption Experiment

The ability of the as-obtained PU/RE nanofibrous membranes to absorb VOCs was tested in a closed container with an air inlet and outlet, as shown in Figure 1. Pristine PU and PU/RE nanofibrous membranes were cut into 8 cm × 8 cm sections, respectively. A rotary pump was used to remove impurities and other contaminants on the fiber surface and container. Firstly, VOCs were thoroughly mixed by each component of 100 μg involving styrene, xylene, chloroform, benzene, and toluene. Next, methanol was used to dilute the solution and to prepare 2 μg of VOCs. Finally, a syringe filled with 2 μg of VOCs was injected into a container that contained PU/RE nanofibrous membranes. In order to make the surface of the nanofibrous membranes more fully contact with VOCs, padded aluminum foil was placed under the nanofibrous membranes as a scaffold. Then the VOCs were absorbed fully by the composite PU/RE nanofibrous membranes for 1 h at room temperature. Afterwards, a nitrogen stream was used to feed the unabsorbed VOCs into a Tenax absorber (Tenax-GR; Japan Analytical Industry, Tokyo, Japan). In this VOCs absorption experiment, the VOCs absorption ability of PU/RE nanofibrous membranes was analyzed by GC/MS (gas chromatography-mass spectroscopy, XEVO TQ-S, Waters Corporation, Milford, CT, USA), Automated Purge & Trap analyzer JTD-505II (Japan Analytical Industry Co., Ltd., Tokyo, Japan), and AGC/MS QP 2010 Plus (Shimadzu, Kyoto, Japan). More specific VOCs analysis methods have been previously shown [18,21].

## 3. Results and Discussion

### 3.1. Physical Properties of Spinning Solutions and Properties of PU/RE Fiber

The electrospinning effect and VOC adsorption capacity of the PU/RE nanofibrous membranes were determined by the physical properties of the spinning solutions and the properties of the PU/RE composite nanofibers. Table 1 shows the physical properties of the spinning solutions and the properties of the PU/RE composite nanofibers. The specific surface area was measured by the N_2_ adsorption-desorption isotherms test via the Brunauer–Emmett–Teller (BET) method. It was found that the viscosity, conductivity and specific surface area increased by increasing the amount of RE powder in the spinning solution. However, the fiber diameter first increased from 489 nm to 524 nm and then decreased from 524 nm to 356 nm when the relative content of the RE powder reached 50 wt. %. The PU nanofiber containing 50 wt. % of the RE powder had the smallest fiber diameter, which was 356 nm. The high conductivity of the spinning solution (0.315 ms/m) led to a better electrospinning effect [21].

### 3.2. Morphological Characteristics of the PU/RE Nanofibrous Membranes

**Figure 2:**
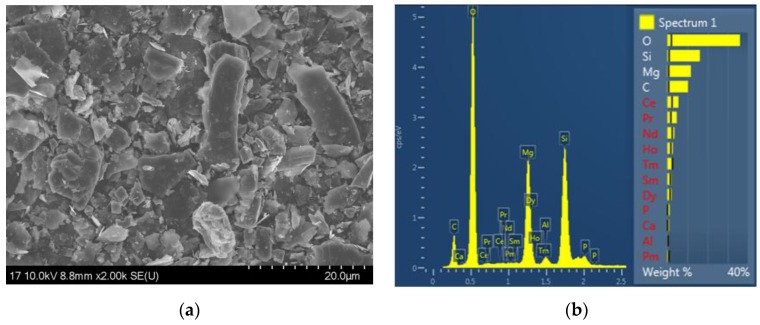
Field emission scanning electron microscopy (FE-SEM) (**a**) and energy-dispersive X-ray spectroscopy (EDX) (**b**) images of the pristine RE powder.

In order to analyze the pristine RE powder, FE-SEM and energy-dispersive X-ray spectroscopy (EDX) images of the pristine RE powder were taken before introducing the nanofibers, which are shown in Figure 2. It can be seen from Figure 2a that the pristine RE powder has very irregular shapes and different sizes. Figure 2b shows the composition and amounts of each element in the pristine RE powder. There were many rare elements in the pristine RE powder, including Ce, Pr, Nd, Ho, Tm, Sm, Dy, and an extremely small amount of Pm. 

**Figure 3:**
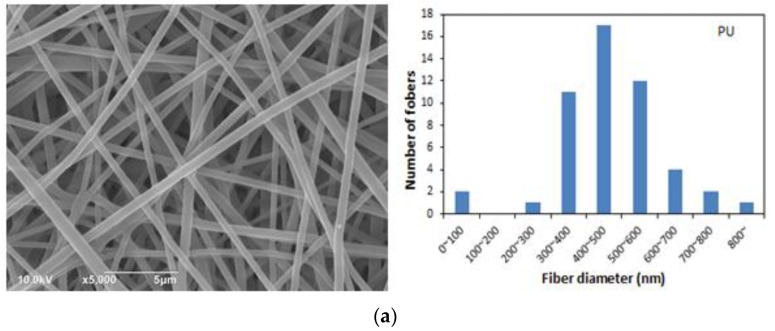

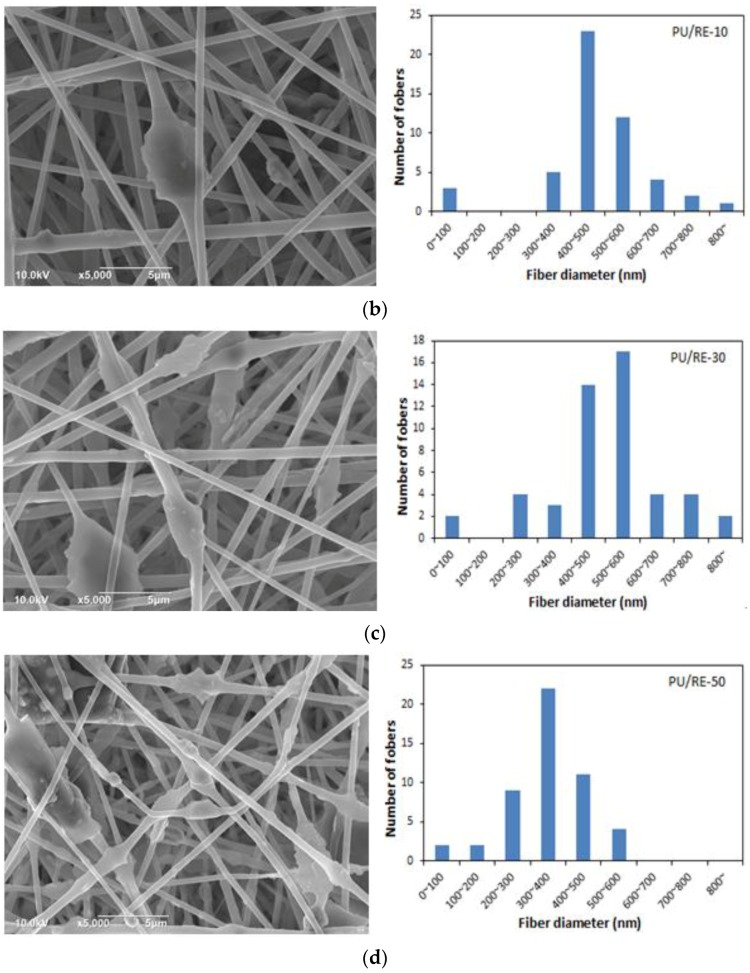
FE-SEM images and diameter distributions of (**a**) polyurethane (PU); (**b**) PU/RE-10; (**c**) PU/RE-30 and (**d**) PU/RE-50.

Figure 3 shows the morphology and the fiber diameter distribution of the composite PU/RE nanofibrous membranes. From Figure 3a–d, it was observed that the changes of the fiber morphology and fiber diameter distribution correlated very closely with the increasing amounts of RE powder in the PU fibers. The pristine PU fibers had smooth surfaces and an average fiber diameter of 489 nm. However, it was found that there were a lot of RE NPs embedded in the PU fibers, and the number of RE NPs increased with an increased concentration of RE powder (Figure 3b–d). In addition, some particle agglomeration existed in Figure 3d because higher concentrations of RE powder cannot be completely dispersed into the spinning solution. During the actual experimental operation, the electrospinning nozzle was blocked two times when the PU/RE nanofibrous membrane with 50 wt. % RE was produced. We also tested other concentrations of PU/RE spinning solutions (PU/RE-55 and PU/RE-60) using the same electrospinning conditions. In these cases, the clogging of the electrospinning nozzle was very serious, and the composite PU/RE nanofibrous membranes could not be manufactured when the higher concentration of RE was added into the PU solution.

Figure 3e–h show the average fiber diameters of the PU, PU/RE-10, PU/RE-30 and PU/RE-50 nanofibrous membranes, which were 489, 513, 524 and 356 nm, respectively. Each of the reported PU/RE nanofibers’ diameters represents an average of 50 random fibers. The fiber diameters increased slightly as the amount of RE powder increased from 0 to 30 wt. %, and then showed a marked decrease when the relative amount of RE powder was 50 wt. %. The PU/RE-50 possessed the smallest average diameter of 356 nm. A probable cause of this may be due to the higher conductivity of the PU/RE-50 spinning solution compared to the other spinning solutions. Based on [21], this result has also been confirmed, as shown in Figure 3e–h. The fiber diameter distribution range of PU/RE-50 nanofibrous membrane was more narrow and concentrated compared with other nanofibrous membranes, and most of them were distributed in the range of 300–500 nm.

### 3.3. Morphological Characteristics of the PU/RE Nanofibrous Membranes

**Figure 4:**
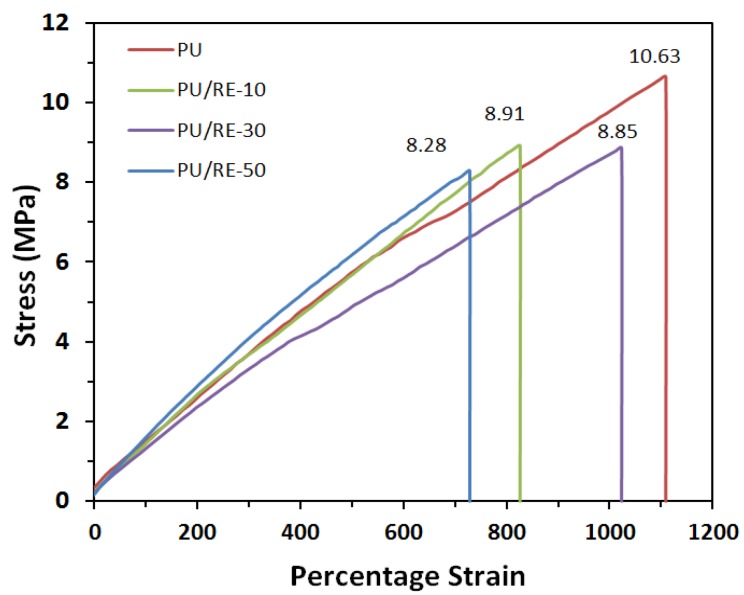
Tensile strength of electrospun PU mats with different amounts of RE.

Nanofibrous filtration membranes exhibit efficient VOC absorption efficiency as well as good mechanical properties, traits which may indicate a potential for commercial application, such as in an air filter installed in a vehicle that can reduce the vehicle’s fuel consumption due to its lighter weight. The mechanical properties of the PU/RE nanofibrous membranes are presented in Figure 4. The tensile strength of each of the composite PU/RE nanofibrous membranes was tested five times to obtain an average value. As shown in Figure 4, the tensile strengths of the PU/RE nanofibrous membranes decreased gradually as the amount of RE powder increased, from 10.63 to 8.28 MPa. The tensile strength of the pristine PU nanofibrous membrane was 10.63 MPa, and it is similar to the research results of Kim et al. [21]. The tensile strength of the PU/RE nanofibrous membranes decreased by only 22.1% when the amount of RE powder reached 50 wt. % compared with the pristine PU nanofibrous membrane. An explanation may be that adding the RE powder to the PU spinning solution led to slight disordering of the fibers as well as the destruction of fibrous regularity during electrospinning. From Figure 3b–d, an obvious disorder of the fibers can be seen as the amount of RE powder in the PU fibers increases. However, while even these disorders and agglomerated particles can decrease the tensile strength of the composite PU/RE nanofibrous membranes, the composite PU/RE nanofibrous membranes can still be fully used as an air filter because their tensile strength is above 8.28 MPa [36,37]. On the other hand, the PU/RE nanofibrous membranes have outstanding mechanical properties and more than a 700% strain.

**Figure 5:**
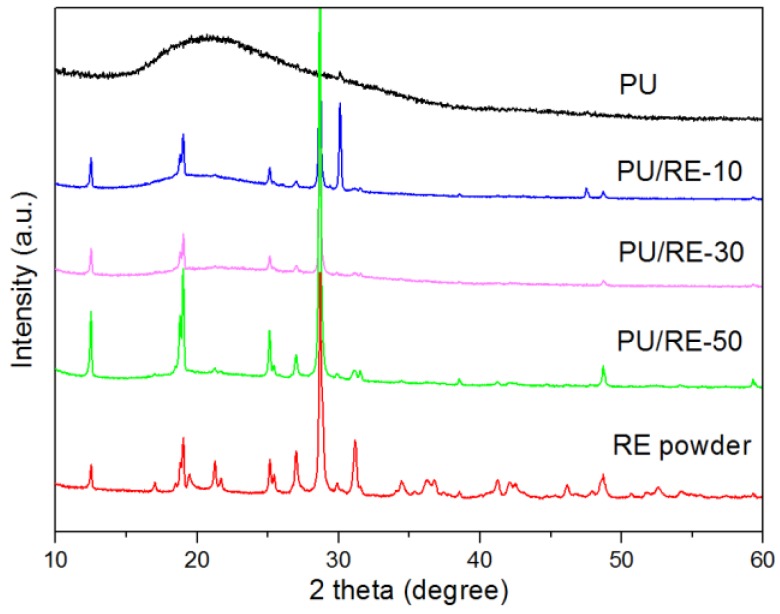
X-ray diffraction (XRD) patterns of different PU/RE nanofibrous mats.

In order to further analyze the RE NPs embedded into the PU fiber and the interactions between the RE powder and PU, the XRD patterns and FT-IR spectra of the PU/RE nanofibrous membranes were investigated. The XRD patterns of the PU nanofibers containing different amounts of RE powder (0 wt. %, 10 wt. %, 30 wt. % and 50 wt. %) are shown in Figure 5. Originally, the RE powder exhibited many characteristic peaks, but after the introduction of RE powder into the PU nanofiber, most of the main XRD peaks emerged and the peak intensity of the composite PU/RE nanofibrous membranes increased significantly when increasing the amount of the RE powder in the PU fibers. It was observed that the RE powder was sufficiently incorporated into the PU NPs in the electrospinning process, and the RE powders were effectively embedded into the PU fibers, which was consistent with the FEM-SEM results. In addition, with the characteristics of the XRD pattern peaks, all the above results demonstrated that a large amount of MgSiO_3_ and CePO_4_ existed in the as-prepared PU/RE fiber mats. The crystal phases of PrPO_4_, Mg_0.5_Ce_2_(PO_4_)_3_, Mg_3_Si_4_O_10_(OH)_2_ were also found in the composite PU/RE nanofibrous membranes, which indicated that they are a main constituent component of Si^4+^, Mg^2+^, Ce^3+^, Pr^3+^ and Nd^3+^ and they have been successfully incorporated into the host lattice [38]. 

**Figure 6:**
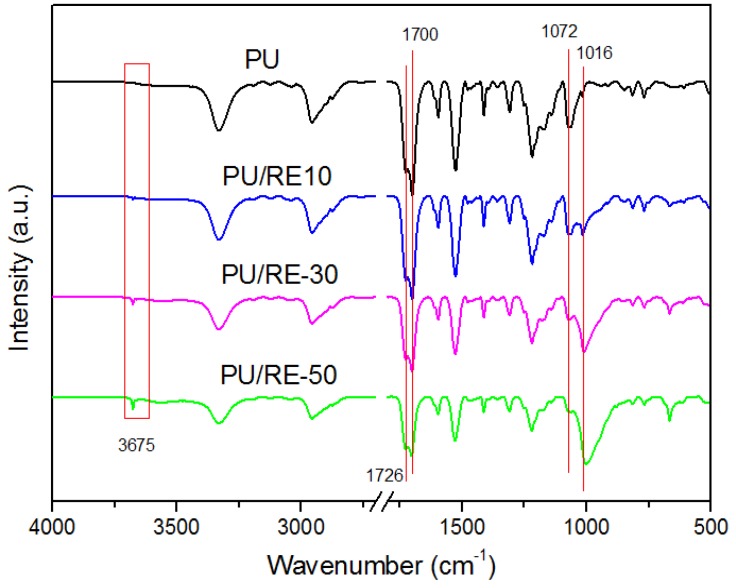
FTIR spectra of different PU/RE nanofibrous mats.

In order to further observe the incorporation characteristics of the RE powder and PU and the phase formation process, the FTIR spectra of the PU nanofibers containing different amounts of RE powder (0 wt. %, 10 wt. %, 30 wt. %, 50 wt. %) are presented in Figure 6. As shown in Figure 6, the FTIR spectrums of the PU/RE composite fibers have no significant changes from 1774 to 2820 cm^−1^. The FTIR spectrum of PU is characterized by the C=O vibration and the N–H vibration at 1726 cm^−1^ and 1700 cm^−1^, respectively. The strong band around 3675 cm^−1^ is associated with the Si-OH bond asymmetric stretching vibrations, and that around 1016 cm^−1^ is the Si–O stretching vibrations, and the peaks (around 3675 cm^−1^ and 1016 cm^−1^) of the composite PU/RE nanofiber gradually increased by increasing the amount of RE powder in the PU fiber. In addition, the band at 1072 cm^−1^ represents the C–O–C vibration [39,40]. The changes were apparent by increasing the amount of RE powder in the PU fiber, which indicates that the addition of the RE powder affected the C–O–C vibration in the PU fiber, showing a weak interaction between the molecules in the RE powder and the PU. This result further shows that the RE powder completely combined with the PU during the electrospinning process.

### 3.4. Absorption Characteristics of the Composite PU/RE Nanofibrous Membranes

**Figure 7:**
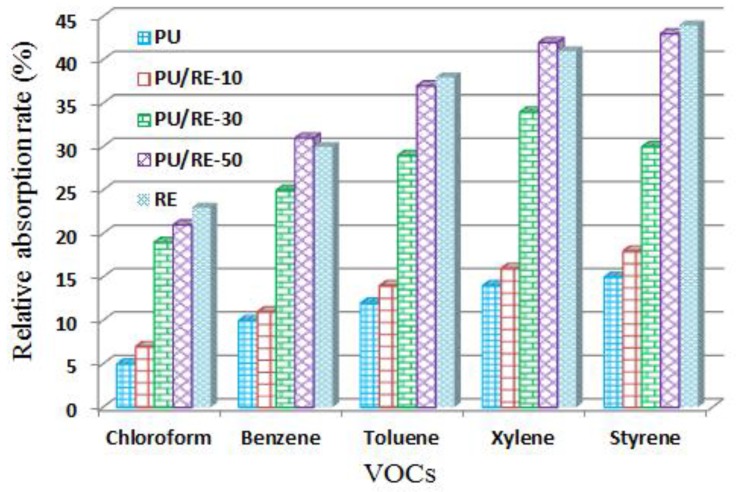
VOC absorption capacity of different nanofibrous mats.

The VOC absorption characteristics of the PU nanofibers containing different amounts of RE powder (0 wt. %, 10 wt. %, 30 wt. %, 50 wt. %) are shown in Figure 7. As shown in Figure 7, it can be seen that the pure PU nanofibrous membrane had a weak VOC absorption capacity (about 10%), while the pure RE powder had a better VOC absorption capacity compared with the pure PU nanofibrous membrane. However, the RE powder will limit its usability. To integrate the merit and eliminate the defect of both PU and RE, the RE-incorporated PU fibers were electrospun before the VOC absorption experiment. The VOC absorption capacity of the composite PU/RE nanofibrous membranes increased as the amount of RE powder in the PU fibers increased. The PU nanofiber containing 50 wt. % RE powder had the highest VOC absorption capacity. In addition, the absorption capacity of the PU nanofiber containing 50 wt. % RE powder increased by 320%, 210%, 208% and 187% compared with that of the pure PU nanofibrous membrane during the absorption of styrene, xylene, toluene, benzene and chloroform, respectively. This may be due to the decreasing fiber diameter of the PU/RE composite fibers as the amount of RE powder increased, which subsequently increased the surface area of the composite fiber, producing a high physical absorption capacity. Furthermore, the higher BET surface area of the PU nanofiber containing 50 wt. % RE powder is 1.64 times greater than the pure PU nanofiber, as shown in Table 1, and it plays an important role in reducing the VOCs [21]. On the other hand, in the chemical adsorption capacity, the RE elements have a certain VOC absorption capacity that has been reported by other researchers [36,37,38,39]. In combination with the physical absorption capacity and the chemical adsorption capacity of the PU/RE nanofibrous membranes, the PU/RE-50 had the highest VOC absorption capacity.

As shown in Figure 7, it can be also seen that the composite PU/RE nanofibrous membranes most easily absorbed styrene, followed by xylene, toluene, benzene and chloroform. Minot et al. [34] and Kim et al. [21] also reported the same result and they suggested that the VOC absorption capacity of the fiber is related to the formation of π-complexes on the fiber surface. The VOC absorption capacity of the fiber becomes stronger as more π-complexes are formed on the fiber surface. On the other hand, the presence of a methyl group would increase the stability of the π-complex [34]. The chemical formulas of styrene, xylene, toluene, benzene and chloroform are C_8_H_8_, C_8_H_10_, C_7_H_8_, C_6_H_6_ and CHCl_3_, respectively. From these chemical formulas it can be seen that styrene contains the most methyl groups, followed by xylene, toluene, benzene and chloroform (no methyl group). Hence, the VOC absorption capacity of the composite PU/RE nanofibrous membranes is the strongest for the absorption of styrene compared with the other VOCs (xylene, toluene, benzene and chloroform).

## 4. Conclusions

In summary, a series of PU nanofibers containing different amounts of RE powder (0 wt. %, 10 wt. %, 30 wt. %, 50 wt. %) with a high VOC absorption capacity can be successfully produced via electrospinning. At RE powder concentrations greater than 50 wt. %, however, the electrospinning nozzle will be seriously blocked. The PU/RE-50 fiber had the smallest fiber diameter, with an average fiber diameter of 356 nm. In addition, this fiber also had outstanding mechanical properties, with an average tensile strength of 8.28 MPa and a percentage strain that was above 700%. Most importantly, it had the highest VOC absorption capacity, more than three times that of the pure PU nanofiber. In addition, the composite PU/RE nanofibrous membranes most easily absorbed styrene, followed by xylene, toluene, benzene and chloroform. This work verified that the PU/RE nanofibrous membranes have a high VOC absorption capacity, and our report also has a certain reference value regarding the study of composite functional nanofibers.

## Figures and Tables

**Table 1:** Physical properties of polyurethane/rare earth (PU/RE) spinning solutions.

Spinning Solutions (PU/RE)	Viscosity (cps)	Conductivity (ms/m)	Fiber Diameter (nm)		BET Surface Area (m^2^/g)
			Distribution	Mean	
PU	379.0	0.172	227–877	489	6.853
PU/RE-10	439.0	0.183	357–966	513	7.147
PU/RE-30	464.2	0.191	262–871	524	7.729
PU/RE-50	504.6	0.315	179–591	356	11.207

BET: Brunauer–Emmett–Teller; PU: PU nanofibers containing 0 wt. % RE powder; PU/RE-10: PU nanofibers containing 10 wt. % RE powder; PU/RE-30: PU nanofibers containing 30 wt. % RE powder; PU/RE-50: PU nanofibers containing 50 wt. % RE powder.
